# Natural History Analysis of 101 Severe Dysplasia and Esophageal Carcinoma Cases by Endoscopy

**DOI:** 10.1155/2017/9612854

**Published:** 2017-03-30

**Authors:** Jin-Wu Wang, Chen-Tao Guan, Li-Li Wang, Ling-Yun Chang, Chang-Qing Hao, Bian-Yun Li, Ning Lu, Wen-Qiang Wei

**Affiliations:** ^1^Department of Pathology, Cancer Hospital of Linzhou, Henan, China; ^2^Department of Cancer Epidemiology, National Cancer Center/Cancer Hospital, Chinese Academy of Medical Sciences, Peking Union Medical College, Beijing, China; ^3^Department of Epidemiology, Cancer Hospital of Linzhou, Henan, China; ^4^Department of Endoscopy, Cancer Hospital of Linzhou, Henan, China; ^5^Department of Pathology, National Cancer Center/Cancer Hospital, Chinese Academy of Medical Sciences, Peking Union Medical College, Beijing, China

## Abstract

*Objectives*. Our research is to realize the natural history from dysplasia to carcinoma and to provide evidence for exploring proper screening intervals. *Methods*. After the onset endoscopy screening, 2093 of the patients participated in the endoscopic follow-up voluntarily. Totally, 101 severe dysplasia and carcinoma cases, either diagnosed in the first endoscopy without treatment or diagnosed in the second endoscopy, were included in our study. We compared the pathologic results of their two endoscopies and calculate the mean and median progression time. *Results*. Of the 39 severe dysplasia cases diagnosed by the onset endoscopy, only 8 progressed to carcinoma. For severe dysplasia cases diagnosed by the follow-up endoscopy, mean progression times are 55.0, 49.8, and 38.0 months and median progression times are 43, 56, and 31 months for esophagitis, mild dysplasia, and moderate dysplasia, respectively. For superficial carcinoma cases diagnosed by the second endoscopy, mean progression times are 76.0, 57.4, and 47.0 months and median progression times are 77, 63, and 35 months for mild dysplasia, moderate dysplasia, and severe dysplasia, respectively. *Conclusions*. Population-based severe dysplasia cases may have much lower carcinoma progression rate than specific-selected ones. The progression time for most enrolled cases seems longer than that of the recent screening protocol recommended.

## 1. Introduction

The data from GLOBOCAN 2012 shows that esophageal cancer was the eighth most common cancer and the sixth most common cause of cancer death, with estimations of 456,000 new cases and 400,000 deaths in 2012 [1]. While in China, it is the sixth most common cancer and the fourth common cause of cancer death, with about 287,000 new cases and 211,000 deaths in 2012 [2], and nearly 95% are esophageal squamous cell carcinoma [3]. From the ratio of mortality to incidence, we can recognize that esophageal cancer is a poor-survival cancer, the age-standardized 5-year relative survival in China is only 20.9% [4]. Since China accounts for about 50% of the cases and deaths of esophageal cancer in the world, the disease burden is quite severe.

Early detection and treatment are an effective way to improve survival and life quality of esophageal cancer patients. As the sensitivity and specificity of endoscopic screening with Lugol's iodine staining become more and more clear [5], its application is gradually expanded in esophageal cancer high-risk areas and the long-term effect of decreasing incidence and mortality has been demonstrated [6]. Dysplasia has been proven to be significant precursor lesions of esophageal cancer [7, 8], and with the increase in dysplasia grades, the carcinogenesis risk increases. So it is extremely necessary to explore its more detailed natural history and progression with a close long-term follow-up.

In China, the current screening protocol indicates that if mild dysplasia (mD) and moderate dysplasia (MD) are found, the patients should be followed up by endoscopy every 3 years and 1 year; if severe dysplasia (SD)/carcinoma in situ (CIS) and superficial carcinoma are found, early treatment should be taken in time; the untreated SD should be followed up every year. But actually there were few specifically designed articles on the progression time of precursor lesions; the screening protocol is more based on experts' experience, rather than convictive researches. What is more, different experts hold different opinions about endoscopy follow-up intervals for different precursor lesion grades. Our study focuses on the progression time from normal/esophagitis, mD, and MD to SD/CIS and superficial carcinoma and tries to explore appropriate endoscopy follow-up intervals for monitoring the development of precursor lesions. However, standard follow-up intervals are always hard to determine because different objectives may require different intervals, not to speak of cost-benefit, compliance, severity of consequence, population characteristics, and so on. So the significance of our study is more on offering meaningful information of lesion progression, rather than determining follow-up intervals arbitrarily according to our research results.

## 2. Materials and Methods

### 2.1. Patients

**Figure 1:**
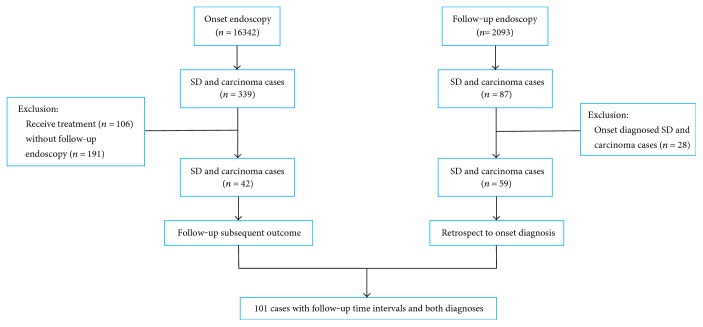
Flow chart of case enrollment.

From 2005 to 2012, the National Cancer Early Detection and Treatment Project was implemented in Linzhou, Henan Province, and the residents from 40 to 69 years old were enrolled to take early screening of esophageal cardiac cancer. Then, from May 2012 to February 2014, 2093 of them took the endoscopic follow-up voluntarily. Totally, 101 SD and carcinoma cases, who had both onset and follow-up endoscopies, were selected into our research, and the treated SD and carcinoma cases after the onset endoscopy were excluded. Of the included 101 cases, 42 SD and superficial carcinoma cases were diagnosed in the onset endoscopy and 58 new SD and superficial carcinoma cases were diagnosed in the follow-up endoscopy, as well as an advanced carcinoma case developed from MD (Figure 1).

### 2.2. Screening Procedure

The two endoscopies were taken after the informed consent was completed and contraindication was excluded. Endoscopy procedure referred to Technology Scheme for Early Detection of Cancer (2011 version) [9]. Lugol's iodine solution was regularly used to stain esophagus, the unstained foci were targeted for biopsy, and regular one biopsy was taken if the unstained region was not evident to avoid the loss of precursor lesions. Biopsy specimens were fixed in 10% buffered formalin, embedded in paraffin, cut into sections, and stained with hematoxylin and eosin. The biopsy slides were read by two local well-trained pathologists, whose diagnosis results were under the sampling quality supervision of the National Cancer Center/Cancer Hospital, Chinese Academy of Medical Sciences. Both pathologists read all of the slides independently, and they would discuss to achieve the consensus of option if their diagnoses were inconsistent. The histopathological criteria of squamous dysplasia is mD, MD, and SD/CIS [7, 10]. Abnormal issues of epithelium present in lower one-third are mD, present in lower two-third and higher one-third are MD, and present in higher two-third or full thickness of the epithelium without invasion are SD/CIS. Superficial carcinoma means that the tumor only invades mucosae or submucosa, without consideration of regional lymph node metastasis or distance metastasis. As the carcinogenesis risk of SD could be very high, our research collects cases focusing on SD and carcinoma. According to the current screening protocol in China, cases above SD were recommended to be cured in time. But maybe for the reason of economy (the endoscopy was free, but the treatment was not), as well as limited awareness of early detection and treatment, not all the local cases would like to be treated immediately. So there existed 39 SD cases and 3 superficial carcinoma cases in our study after the onset endoscopy screening.

### 2.3. Design and Statistics

Our research was based on the National Cancer Early Detection and Treatment Project and not independently designed. From 2012 to 2014, the previous screened participants were encouraged to be endoscopically followed up voluntarily, the second endoscopy was cost-free, and the time was not limited. During the second endoscopy process, we firstly confirmed that the lesions were the same with the onset-found lesions. Then, for the 101 cases, we followed up the subsequent development of onset-diagnosed SD and superficial carcinoma but not cured ones and retrospected to the onset diagnosis of follow-up-diagnosed SD and carcinoma ones. Follow-up endoscopy time minus onset endoscopy time was used to get the time interval and calculated mean progression time and median progression time of different lesion grades.

## 3. Results

The baseline information is shown in Table 1. Of the 39 SD cases diagnosed by the onset endoscopy, 17 remained stable and 14 downgraded with different degrees (Table 2). The other 8 SD cases progressed to superficial carcinoma with a mean time of 33.8 months and a median time of 30 months. Of the 3 onset-diagnosed superficial carcinoma cases, 2 progressed to advanced carcinoma in 76 and 87 months and the other remained stable.

Of the 48 follow-up-diagnosed SD cases, 5 developed from esophagitis with a mean time of 55.0 months and a median time of 43 months, 16 developed from mD with a mean time of 49.8 months and a median time of 56 months, and 27 developed from MD with a mean time of 38.0 months and a median time of 31 months. Of the 10 follow-up-diagnosed superficial carcinoma cases, 5 developed from mD with a mean time of 76.0 months and a median time of 77 months and 5 developed from MD with a mean time 57.4 months and a median time of 63 months (Table 3). One MD case progressed to advanced carcinoma in 75 months.

**Figure 2:**
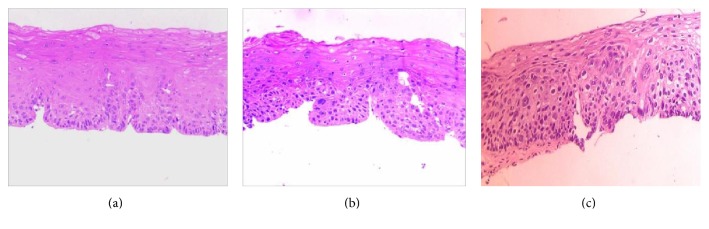
Pathological progression micrographs of case 1: (a) mild dysplasia in October 2009; (b) moderate dysplasia in April 2013; (c) severe dysplasia in August 2015.

**Figure 3:**
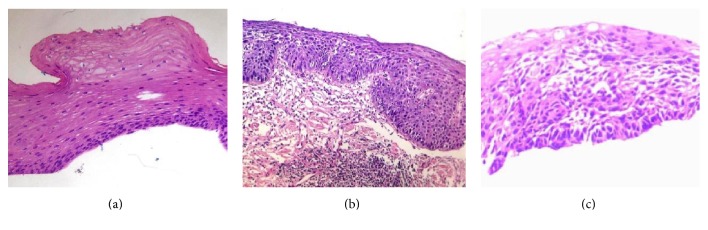
Pathological progression micrographs of case 2: (a) mild dysplasia in July 2007; (b) moderate dysplasia in December 2013; (c) severe dysplasia in August 2015.

**Figure 4:**
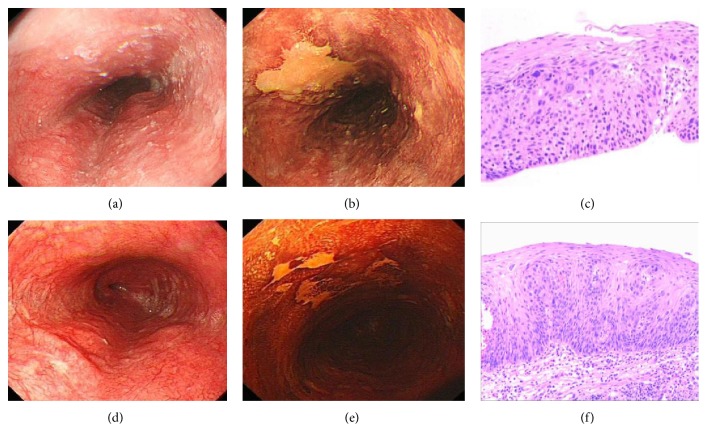
Pathological downgrading macrographs and micrograph of case 3 from severe dysplasia to mild dysplasia: (a) severe dysplasia macrograph under white light in September 2010; (b) severe dysplasia macrograph under Lugol's iodine staining in September 2010; (c) severe dysplasia micrograph in September 2010; (d) mild dysplasia macrograph under white light in May 2013; (e) mild dysplasia macrograph under Lugol's iodine staining in May 2013; (f) mild dysplasia micrograph in May 2013.

All the voluntary patients only have one follow-up endoscopy before February 2014, which was the cutoff date of our study. In order to show the whole malignant progression of dysplasia, pathological micrographs of 2 cases progressed from mD and MD to SD are displayed in Figures 2(a), 2(b), and 2(c) and Figures 3(a), 3(b), and 3(c). But the time of third micrographs was August 2015, which was out of the study cutoff date.  Figure 4 is the pathological downgrading macrographs and micrograph of case 3 from severe dysplasia (Figures 4(a), 4(b), and 4(c)) to mild dysplasia (Figures 4(d), 4(e), and 4(f)).

## 4. Discussion

SD and CIS have been proven that they have the similar subsequent cancer risk and the diagnostic distinction should be abandoned [7]. The current screening protocol in China also suggests that SD and CIS cases should be cured with the same clinical procedure. However, our study showed that onset-diagnosed SD cases existed the downgrade phenomena. Based on the current understanding of SD, few SD cases can regress to low grade even though this is also a possibility. The main explanation about these phenomena should be that biopsy may move those smaller than 1 cm lesions, and the onset endoscopy means a treatment, which indirectly supports that endoscopic mucosal resection (EMR) is a feasible way to cure precursor lesions. The other explanation is that the downgraded lesions may actually be reactive hyperplasia but not dysplasia, so diagnosis merely based on pathological morphology may have limitations; more accurate diagnosis with biomarkers may be needed in the future. Meanwhile, 17 of the onset-diagnosed SD cases remained stable and only 8 cases progressed to superficial carcinoma, which means that only about 20% (8/39) of SD cases progressed to superficial carcinoma. Even though SD is a high-risk precursor lesion stage, not all the SD cases will progress, some cases may even remain stable for a long time. For those stable or downgraded SD patients, immediate treatment with therapeutic endoscopy may not be necessary.

Of the 48 cases progressed to SD, the proportions increase from esophagitis and mD to MD (5/48, 16/48, and 27/48); so do the 18 cases progressed to superficial carcinoma, developed from mD, MD, and SD, and also nearly had the ascending trend (5/18, 5/18, and 8/18). Besides, with the increase in the grades, both mean progression time and median progression time presented the shortening trends (Table 3), except for the median time of esophagitis progressing to SD. But the time intervals of 5 SD cases progressed from esophagitis were 47 to 76 months. As their progression time all above 3.5 years, we consider them new esophageal cancer precursor lesions, which is similar with the concept of new gastric cancer, proposed by Raftopoulos et al. [11]. So the above proportion trend and progression time trend not only indicate that dysplasia is important path to carcinoma but also confirm the consecutive process of esophageal cancer progression: normal/esophagitis → mD → MD → SD/CIS → superficial carcinoma → advanced carcinoma.

Excessively frequent endoscopy follow-up not only wastes medical resource and leads to overtreatment but also lowers down the compliance, while overlong follow-up intervals may cause the unacceptable disease progression. One previous study conducted in high-risk areas of China suggested that baseline cell hyperplasia and mD should be endoscopically followed up every 5 years and MD is every 3 years [12]. While another study advised every 2- to 3-year interval for baseline cell hyperplasia and mD and every half-year interval for those MD or above MD cases [13], there exists big difference on determining follow-up time intervals.

In our research, according to the progression time of SD cases developed from mD (mean time, 49.8 months; median time, 56 months) and MD (mean time, 38.0 months; median time, 31 months), we think every 4.5-year and 3-year interval for mD and MD, respectively, will find nearly half of them have progressed to SD. Similarly, according to the progression time of superficial carcinoma cases developed from mD (mean time, 76.0 months; median time, 77 months), MD (mean time, 57.4 months; median time, 63 months), and SD (mean time, 47.0 months; median time, 35 months), we think every 6.5-year, 5-year, and 3-year interval for mD, MD, and SD, respectively, will find nearly half of them have progressed to superficial carcinoma.

As cancer is often a poor-prognosis disease, the best way against it is to avoid its occurrence and block it in the precursor lesion period. The goal of endoscopy screening is to find and cure high-risk precursor lesions or superficial carcinoma in time, as well as follow up the low-risk precursor lesions. The phenomena such as MD progressed to advanced carcinoma in our study are unacceptable. More strictly, we could better avoid any carcinoma including the superficial carcinoma. So for the objective of nonprogression to carcinoma, we would like to take 3.5 years and 2.5 years as the follow-up intervals of mD and MD, respectively, which are shorter than the earliest progression time (44 months and 31 months) to superficial carcinoma. For those refusing treated SD patients due to limited health resources as well as awareness of early detection and treatment, close endoscopy follow-up can be taken every year, and the patients should be cured immediately if the lesions are progressing with presenting the following three situations: (1) lesion areas expand or the surficial abnormal morphology becomes severer, (2) the degree of iodine unstaining increases, and (3) the pathological results show exacerbation results.

Of course, we do not deny that the above recommendations about endoscopy follow-up intervals are relatively conservative when standing at the aspect of public health, because the present endoscopy therapies such as EMR and endoscopic submucosal dissection (ESD) can well cope with superficial carcinoma. But from the clinical perspective, we cannot exclude the risk of fast progression from low-risk precursor lesions to advanced carcinoma, just like the specific MD case in our study. However, the other notable thing is that the above descriptions and inference about follow-up intervals are based on the hypothesis of certain lesion progression. Actually, in our study, only about 20% of untreated SD cases have progressed during the two endoscopy periods. This may indicate a lower progression rate of precursor lesions detected in population-based screening (20.5% versus 73.9%), when compared with that in the previous specific-selected case study [7]. Since all the onset-diagnosed mD and MD cases in our research were retrospected through the follow-up-diagnosed SD or carcinoma cases, we could not calculate the progression rate of mD and MD, but we could image that it must be lower than 20%. If their low progression rates were confirmed in the further study, we would have more reasons to revise the current controversial screening protocol.

Now, no matter for the progression rates or follow-up intervals, the results in our study seem to be doubting the applicability of the current esophageal cancer screening protocol in China. The present endoscopy follow-up may be used too frequently and lack of more accurate population stratifications for intervention. While our results and conclusions should not be easily extended, but these should also be considered a clear signal that the esophageal cancer follow-up intervals by endoscopy need further research to determine. Firstly, our research is not a strict cohort-designed study, it was based on the National Cancer Early Detection and Treatment Project, and we analyze the existing data. As the secondary endoscopy is voluntary, the compliance is very low and volunteer bias could not be avoided. We only included SD and carcinoma cases who had both endoscopies. Besides, the onset-diagnosed SD and carcinoma cases with timely treatment were excluded. So our population-based conclusions were merely come from untreated SD and carcinoma cases. Secondly, also because the endoscopy is voluntary, our research lacks time-fixed and continuous follow-up to observe the whole carcinogenesis procedure. Lastly, since the sample size of our study is small, the probative force is limited. Larger-sample-size research based on populations with perfect design needs to be conducted to confirm our results in the future.

Comprehensive speaking, our research find that population-based severe dysplasia cases may have much lower carcinoma progression rate than the previous specific-selected ones, and there exist the phenomena of remaining stable and even maybe downgrading for some SD cases. The progression time of most mD, MD, and SD cases also seems longer than the protocol-recommended follow-up intervals. So the current screening protocol may need more careful demonstration and deliberation.

## Figures and Tables

**Table 1:** Baseline information of 101 SD and carcinoma cases.

	Number	Percentage (%)
Gender		
Male	50	49.5
Female	51	50.5
Age group		
40–49	9	8.9
50–59	55	54.5
60–69	37	36.6
Smoke		
No	78	77.2
Yes	23	22.8
Alcohol		
No	98	97.0
Yes	3	3.0
Other disease history		
No	83	82.2
Yes	18	17.8
Family cancer history		
No	56	55.4
Yes	45	44.6

**Table 2:** Downgraded results of 14 onset-diagnosed SD cases.

Progression	Number	Follow-up intervals (month)
SD → MD	4	17, 28, 32, 68
SD → mD	6	23, 32, 56, 70, 75, 76
SD → esophagitis	4	32, 75, 76, 76

**Table 3:** Progression time of SD and superficial carcinoma.

Onset diagnosis	Follow-up diagnosis							
	SD				Superficial carcinoma			
	Number	Time intervals^†^	Mean^†^	Median^†^	Number	Time intervals^†^	Mean^†^	Median^†^
Esophagitis	5	43–76	55.0	43	—	—	—	—
mD	16	13–77	49.8	56	5	44–98	76.0	77
MD	27	19–86	38.0	31	5	31–75	57.4	63
SD	17	5–89	33.8	30	8	24–86	47.0	35

^†^The unit is month.
